# Diagnostic uncertainty of steroid-modified Marburg's variant of multiple sclerosis even at autopsy: A case suggesting lymphoma and related myelin loss

**DOI:** 10.1016/j.ensci.2024.100515

**Published:** 2024-07-11

**Authors:** Akira Hanazono, Keita Yasuda, Hinako Shimada, Yoshiko Takahashi, Homare Funasaka, Yui Sanpei, Masashiro Sugawara

**Affiliations:** a Akita University Graduate School of Medicine, Department of Neurology, Japan; b Department of Neurology, Akita City Hospital, Japan; c Department of Neurology, Omagari Kousei Medical Center, Japan

**Keywords:** Mass effect, Central nervous system lymphoma, Sentinel lesion, Myelin loss, Leptomeningeal metastasis

## Abstract

MS (multiple sclerosis) has specific criteria to avoid misdiagnosis. However, the Marburg variant of MS is so fulminant that initial axonal damage and other atypical observations have been allowed in past reports. We present a 74-year-old autopsy case with a vanishing tumor after steroids and radiation therapy, which was pathologically diagnosed as a Marburg variant with initial axonal loss. The case displayed radiological lymphoma-like observations: mass effects protruding to the lateral ventricle, fused extension from the choroid plexus to white matter with C opening sign, a growing lesion from the skull dura mater, high in diffusion-weighted imaging and low in apparent diffusion coefficient on magnetic resonance imaging (MRI) suggesting high cell density lymphoma. In addition, clinical manifestations were atypical for MS: upper limb monoplegia without ipsilateral lower limb involvement, pleocytosis over 50 cells/μL, and class 3 cytological abnormality in cerebrospinal fluid. However, at autopsy following steroids and radiation therapy, there were no lymphoma-like lesions, such as mass effects, fused extensive lesions, masses on the skull dura mater, or high cell density lesions. Instead, there were only myelin losses corresponding to the MRI lesions, highlighting the potential for contamination by other diseases in steroid-modified Marburg's variant of multiple sclerosis, possibly due to lymphoma, even at autopsy.

## Highlights

• A case challenging the certainty of the steroid-modified Marburg variant of multiple sclerosis even at autopsy.
• Steroid-mediated disappeared lymphomas and tumor-mediated myelin loss were likely etiology.
• A certain number of Marburg variants have been contaminated by lymphoma due to the steroids and overdependence on histopathology.

## 1 Introduction

Multiple sclerosis (MS) is defined by primary myelin disturbance without initial axonal damage. It should be strictly differentiated from axonal degenerative disease, which secondary leads to myelin degeneration [1]. Without strict exclusion, MS runs the risk of being contaminated with other diseases, as emphasized in the successive McDonald's diagnostic criteria [2].

On the other hand, since the first report by Marburg in 1906, fulminant and often fatal forms of MS have been reported, primarily based on pathological similarities to MS. This MS variant even allows for initial axonal damage [[3], [4], [5]], bypassing the aforementioned pathological definition of demyelinating disease [1]. With no criteria, all fulminant diseases with some white matter affection could be diagnosed with the Marburg variant of MS. Thus, manifestations of Marburg variants differ in each report [3,4,6] and there were no typical radiological features. Nonetheless, rapid brain stem involvement with early dysphagia and the fatal clinical course seemed to be frequent observations [[3], [4], [5], [6]]. Some histopathology were similar to MS with periventricular lesions, sharply marginated lesions with macrophagic infiltration, and inflammations of perivascular inflammation [3,4]. However, pathologies were affected by corticosteroids [4] and some cases even lacked pathological evaluations [6]. Differential diagnosis of the Marburg variant was acute disseminated encephalomyelitis, Balo's concentric sclerosis, Schilder's diffuse sclerosis, and neuromyelitis optica spectrum disorders [[4], [5], [6]]. Recent reports emphasized lymphoma as the important mimic [7,8].

In this context, we report the steroid-modified autopsy case with a pathologically-diagnosed MS with initial axonal loss, despite the clinically and radiologically lymphoma-like manifestations. This discrepancy cautioned the high probability of misinvolvement of other diseases even at autopsy in the Marburg variant.

## 2 Case presentation

**Fig. 1:**
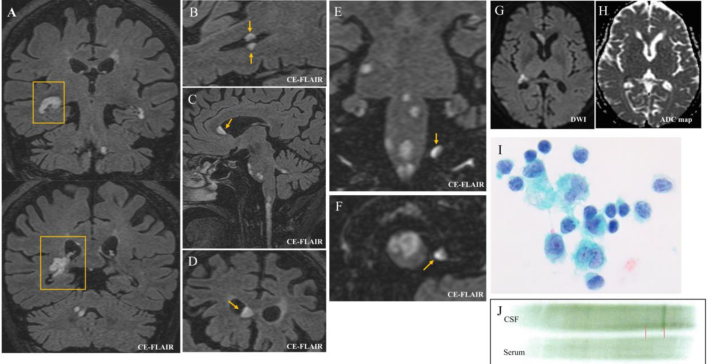
CE-FLAIR MRI of the coronal section shows fused, clustered lesion with the C opening sign arising from choroid plexus (A), and several lesions protrude to the lateral ventricle (A, B, C, D). A lesion growing from the dura mater on the skull, which is not even the brain parenchyma, at the coronal and axial view (E, F). All lesions are high in DWI and low in ADC map (G, H). CSF shows class 3 in cytology, unlike MS (I), and oligoclonal band with strong single band (J).

**Fig. 2:**
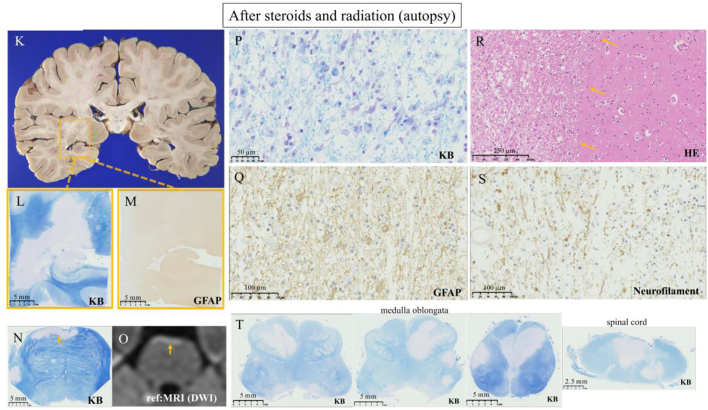
In pathology, there is no mass effect protruding to the lateral ventricle, or mass growing from the dura mater on the skull observed on MRI (K). KB and GFAP staining shows selective myelin damage without astrocytic involvement. Some astrocytes show enlargement (L, M, Q). The KB-unstained lesion is completely matched to the DWI-high lesion (with low ADC map lesion) on MRI (N, O). Myelin was phagocyted (P). The lesion is sharply marginated (R, yellow arrows). Initial axonal damages with swelling are shown in neurofilament staining (S). Most of the lesions of KB-unstained lesions appear to arise from the brain surface, fourth ventricle, or central canal (L, N, T). (For interpretation of the references to colour in this figure legend, the reader is referred to the web version of this article.)

A 74-year-old female with a history of diabetes mellitus (DM) experienced two weeks of headache, appetite loss, and fatigue with unknown etiology. Then, the neurological onset was monoplegia of the right upper limb without ipsilateral lower limb involvement. About a week later, the patient developed tetraplegia, leading to hospitalization. Vital signs, including body temperature, were within the normal range. Magnetic resonance imaging (MRI) with contrast-enhanced fluid-attenuated inversion recovery (CE-FLAIR) showed several extending fused lesions appearing to arise from choroid plexus to the white matter of lateral ventricles with lymphoma-suggesting “C opening sign” (Fig. 1. A, boxes). The surface lesions showed mass effects protruding to the lateral ventricle (Fig. 1. B, C, D). Notably, there was a lesion growing from the skull dura mater, which was even the outside the brain parenchyma (Fig. 1. E, F). All lesions exhibited high intensity on diffusion-weighted imaging (DWI), and low intensity on apparent diffusion coefficient (ADC) with mass effect (Fig. 1. G, H, Fig. 2. O). Laboratory testing showed a mild elevation of soluble interleukin 2 receptor (sIL2R) (515 U/mL), along with elevated transaminase, and hyperglycemia due to DM. Other tests to explain brain lesions were all within normal range, including the anti-myelin oligodendrocyte glycoprotein antibody, anti-aquaporin 4 antibody, anti-neutrophil cytoplasmic antibody, anti-nuclear antibody, angiotensin-converting enzyme, or tumor markers. Paraneoplastic syndrome and related antibodies were not evaluated due to the tumor-like mass effects on MRI. Cerebrospinal fluid (CSF) showed lymphocytic pleocytosis with elevated protein and a relatively decreased glucose (initial/final pressure 11/9.5 cm H_2_O, all mononuclear 54 cells/μL, protein 59 mg/dL, glucose 81 mg/dL, serum glucose for reference 194 mg/dL), and cytology showed class 3 without mitosis, suggestive of reactive lymphocyte (Fig. 1. I). CSF also showed an oligoclonal band consisting of a single strong band with an extremely weak band (which appeared as if “mono”clonal band) (Fig. 1. J). Whole-body computed tomography identified a small right thyroid papillary carcinoma (< 1.0 cm diameter), but it was considered an unlikely cause of central nervous system (CNS) lesions due to its static nature and absence of lymph node metastasis. In a few days after admission, lymphoma emerged as a most likely differential diagnosis, but CSF flow cytometry (requiring additional lumber puncture), brain biopsy, or empiric chemotherapy were not available, because of the worsening severe dysphagia, a drowsy state, and the patient's refusal of additional invasive procedures. Given these manifestations suggesting some malignancy like lymphoma and the patient's will, palliative radiation including whole brain and upper spinal cord (30 Gray/ 10 Fractions), steroids (dexamethasone 3.3 mg/day for 15 days), and intravenous morphine was administered. Following treatment, the patient's condition initially stabilized, but she eventually succumbed to severe aspiration pneumonia with worsening dysphagia and consciousness. In contrast to clinical diagnosis, histopathological observations at autopsy could not detect extending fused lesions with mass effect on MRI, such as malignancy (including lymphoma and thyroid carcinoma), infection like tuberculosis, or any granuloma. Instead, all brain and spinal cord lesions were pathologically diagnosed as Marburg variant of MS, with sharply margined myelin loss (Fig. 2. K, L, N, R), myelin phagocyting (Fig. 2. P), intact astrocytes with minor enlargement (Fig. 2. M, Q), and initial axonal damage despite demyelinating disease (Fig. 2. S). In the lesion of myelin loss, no inflammatory cells were detected. All lesions of myelin loss were completely matched with DWI-high (and ADC-low) intensity lesions (Fig. 2. N, O). In addition, most of the lesions appeared to grow from the brain surface, lateral and fourth ventricles, or spinal central canal (Fig. 2. L, N, T), appearing as if via-CSF dissemination of the lesion.

## 3 Discussion

In histopathology, the final diagnosis of this case was negative for any tumors, and there is no contradiction for the MS, because the clinical course was fulminant and the Marburg variant historically allowed this kind of axonal damage. However, from a clinical/radiological perspective, this case was inconsistent with typical MS.

Firstly, MRI showed intraventricular lymphoma-like manifestations [[9], [10], [11]]. CE-FLAIR showed the lymphoma-suggestive “C opening sign” [12] and fused, clustered lesions from choroid plexus to the white matter at the lateral ventricle [9]. There were mass effects of ventricle surface protruding to lateral ventricles. Notably, a mass appeared to grow from dura mater on the skull, situated even outside the brain, and this decisive observation could not be detected at autopsy. All the lesions were consistently high in DWI and low in ADC, suggesting high cell density lymphoma [13], distinct from the temporal and localized high DWI/low ADC characteristic of acute MS lesions [14]. At autopsy, lymphoma-like lesions on MRI, like mass effects, fused extensive lesions, mass on the skull dura mater, or high cell density lesions, were undetectable. Instead, all MRI lesions completely corresponded to areas of myelin loss (Fig. 2: N, O), suggesting tumor-mediated demyelination. Secondly, clinical manifestations were also inconsistent with MS. Upper limb monoplegia (right arm onset) without ipsilateral lower limb involvement was an unlikely symptom of MS, because MS affects white matter, and if the upper limb is demyelinated, the ipsilateral lower extremity is usually also affected, so lower limbs have been the major concern in MS [15]. This case was so severe that confinement of the upper limb pyramidal tract was unlikely. On the other hand, the cytology showed class 3 abnormalities that could not observed in typical 541 MS patients [16], and pleocytosis over 50 cells/μL is also atypical in MS [16]. Based on these reasons from radiological and clinical aspects, it is reasonable to conclude that this case was far from MS including the Marburg variant.

We hypothesize lymphoma as the fundamental etiology because the most frequent cause of vanishing tumors especially in steroid-using cases is lymphoma [17,18]. In addition, tumor-related demyelination pathology is mostly due to lymphoma, called “sentinel lesion” [10,17,18]. Furthermore, as noted above, all MRI features were consistent with lymphoma, and these radiological distributions resembled the pattern of lymphoma's dissemination through the ventricle surface via CSF [[9], [10], [11],^13^,^19^].

The major issue in the present case is the “negative” result in pathology. Although histopathological evaluations are the most reliable for diagnoses, brain biopsy is more invasive, and it might be avoided especially in patients with deteriorating general status like the Marburg variant. In addition, cases with assumed treatable demyelinating diseases like MS, or terminal stage requiring palliative care might be treated by steroids antecedently, which increases the probability of “vanishing tumor” (mainly lymphoma) [17,18]. On the other hand, an autopsy is regarded as the gold standard/final diagnosis and determines the “negative” case [20] (as if diagnostic testing with 100% sensitivity). Furthermore, “negative” studies are usually unpublished [21]. Therefore, it is an inevitable pitfall for pathology to recognize vanishing tumors with sentinel demyelinating lesions after steroid use as demyelinating disease or malignancy-negative cases, even if clinical and radiological manifestations are significantly inconsistent with MS. The allowance of initial axonal loss in Marburg's variant despite demyelinating disease is exacerbating this pitfall. In addition, unlike the Marburg variant, diagnosable cases of vanishing tumors were confined to slow-progressing or surviving cases because it requires a time course to confirm recurrence [10,17,18].

Given these risks for contaminations by different diseases in pathology, and the younger age distribution of typical MS [18], elderly patients with a fulminant course exhibiting initial axonal loss, as in our case, should not be easily diagnosed as MS. Although we are not saying that all Marburg variant is related to lymphoma, recent therapeutic approaches to the Marburg variant are simultaneously lymphoma-effective drugs (like cyclophosphamide or anti CD20 antibody) [6], and the contaminations by lymphoma in Marburg variant are more inevitable.

## 4 Conclusion

Despite the pathological consistency with MS at autopsy, clinical and radiological observations were significantly inconsistent with MS and rather suggested vanishing tumors like lymphoma and tumor-mediated myelin loss. We should avoid overdependence on pathology, especially in steroid-modified cases, because the Marburg variant is originally a vague disease entity.

## CRediT authorship contribution statement

**Akira Hanazono:** Writing – original draft, Software, Resources, Project administration, Investigation, Data curation, Conceptualization. **Keita Yasuda:** Writing – review & editing, Data curation, Conceptualization. **Hinako Shimada:** Writing – review & editing, Conceptualization. **Yoshiko Takahashi:** Writing – review & editing, Conceptualization. **Homare Funasaka:** Writing – review & editing. **Yui Sanpei:** Writing – review & editing, Methodology, Conceptualization. **Masashiro Sugawara:** Writing – review & editing, Validation, Supervision, Project administration, Methodology, Conceptualization.

## Declaration of competing interest

The authors declare that they have no conflicts of interest.
